# TRAF6 Is Essential for Maintenance of Regulatory T Cells That Suppress Th2 Type Autoimmunity

**DOI:** 10.1371/journal.pone.0074639

**Published:** 2013-09-13

**Authors:** Go Muto, Hitoshi Kotani, Taisuke Kondo, Rimpei Morita, Sanae Tsuruta, Takashi Kobayashi, Hervé Luche, Hans Joerg Fehling, Matthew Walsh, Yongwon Choi, Akihiko Yoshimura

**Affiliations:** 1 Department of Microbiology and Immunology, Keio University School of Medicine, Tokyo, Japan; 2 Japan Science and Technology Agency (JST), CREST, Tokyo, Japan; 3 Department of Infectious Disease Control, Faculty of Medicine, Oita University, Idaigaoka, Hasama, Yufu-shi, Oita, Japan; 4 Institute of Immunology, University Clinics, Ulm, Ulm, Germany; 5 Department of Pathology and Laboratory Medicine, University of Pennsylvania Perelman School of Medicine, Philadelphia, Pennsylvania, United States of America; New York University, United States of America

## Abstract

Regulatory T cells (Tregs) maintain immune homeostasis by limiting inflammatory responses. TRAF6 plays a key role in the regulation of innate and adaptive immunity by mediating signals from various receptors including the T-cell receptor (TCR). T cell-specific deletion of TRAF6 has been shown to induce multiorgan inflammatory disease, but the role of TRAF6 in Tregs remains to be investigated. Here, we generated Treg-specific TRAF6-deficient mice using Foxp3-Cre and TRAF6-flox mice. Treg-specific TRAF6-deficient (cKO) mice developed allergic skin diseases, arthritis, lymphadenopathy and hyper IgE phenotypes. Although TRAF6-deficient Tregs possess similar *in vitro* suppression activity compared to wild-type Tregs, TRAF6-deficient Tregs did not suppress colitis in lymphopenic mice very efficiently due to reduced number of Foxp3-positive cells. In addition, the fraction of TRAF6-deficient Tregs was reduced compared with wild-type Tregs in female cKO mice without inflammation. Moreover, adoptive transfer of Foxp3 ^+^ Tregs into Rag2^-/-^ mice revealed that TRAF6-deficient Tregs converted into Foxp3^-^ cells more rapidly than WT Tregs under lymphopenic conditions. Fate-mapping analysis also revealed that conversion of Tregs from Foxp3^+^ to Foxp3^-^ (exFoxp3 cells) was accelerated in TRAF6-deficient Tregs. These data indicate that TRAF6 in Tregs plays important roles in the maintenance of Foxp3 in Tregs and in the suppression of pathogenic Th2 type conversion of Tregs.

## Introduction

A variety of autoimmune and allergic disease pathologies are caused by the immune responses to “self”, environmental non-microbial antigens and infectious agents. Regulatory T cells (Tregs), which are characterized by expression of the Forkhead transcription factor, Foxp3, play an indispensable role in immunological tolerance, protecting the host from excessive immune responses. Foxp3 plays an essential role in the suppressive function of Tregs, and Foxp3 deficiency causes a multi-organ autoimmune disease, which can be observed in the *scurfy* mouse and in patients with immunodysregulation polyendocrinopathy enteropathy X-linked syndrome (IPEX) [1,2]. Foxp3 induction in natural Tregs (nTregs) occurs *in vivo* during thymic differentiation, under the influence of relatively high avidity interactions of the T-cell receptor (TCR) with self-antigens [3]. Various transcription factors, including c-Rel, Smad2/3, and Runx1 have been identified to be important for Treg induction by transactivating the *Foxp3* promoter and/or enhancers [4,5]. In addition, we have shown that the NR4a family of transcription factors, which could be a direct sensor of TCR strength, are essential for Treg development in the thymus [6].

Although the Treg suppression mechanism is now well characterized [7], the molecular mechanisms of Treg development and maintenance remain to be clarified. nTregs have been shown to convert to effector helper T cells such as Th1, Th17 and follicular helper T (Tfh) cells [8,9]. Most Tregs retain high Foxp3 expression following the adoptive transfer into recipients with a nonpathogenic setting. However, substantial fractions of Tregs were found to lose Foxp3 expression and begin to produce interleukin (IL)-2 and interferon-gamma (IFN-γ) under lymphopenic conditions [8]. Additionally, several recent studies have demonstrated that in the inflammatory setting of autoimmunity, there is a loss of Foxp3 during inflammatory responses [10,11]. These exFoxp3 cells which lost Foxp3 expression among Foxp3^+^ Treg cells develop an effector-memory phenotype, produce pathogenic cytokines, and may be involved in triggering the development of autoimmunity. In contrast, recent study by Miyao et al. clearly denied Treg reprogramming, however, they showed that a few Treg cells transiently lose Foxp3 expression, but robustly re-expressed Foxp3 and suppressive function upon activation [12]. However, it is still an open question how such stability and/or re-expression of Foxp3 in Tregs are regulated. We have reported that SOCS1, an inhibitor of cytokine signaling, plays an essential role in suppressing the conversion of Tregs to exFoxp3 cells [13]. The signals to maintenance of stability of Tregs remained to be clarified.

Tumor necrosis factor receptor (TNFR)-associated factor (TRAF) 6 transduces signals from several members of the TNFR superfamily and the TLR⁄ IL-1R family to activate the transcription factors NF-kB and AP-1 [14]. It has been also shown that TRAF6 is required for NF-kB activation, which is induced in response to TCR stimulation by binding to mucosa-associated lymphoid tissue (MALT) 1 in Jurkat T cells [15]. Using a mouse model of T cell–specific TRAF6 deficiency, we previously demonstrated that TRAF6 in CD4^+^ T cells is critical for induction of peripheral tolerance and anergy [16]. TRAF6-deficient effector T cells were resistant to Tregs through an enhanced PI3 kinase pathway [16]. In addition, Motegi et al reported that TRAF6-deficient T cells were hypersensitive to IL-2 because the binding of TRAF6 to the IL-2 β-chain negatively regulates IL-2-induced Jak1 activation [17].

This study was undertaken to clarify the role of TRAF6 in the stability and suppressive function of Tregs. We observed Th2-prone autoimmune phenotypes in Treg-specific *TRAF6* conditional knockout (cKO) mice, suggesting defective Treg functioning in these mice. The defective suppression activity of TRAF6-deficient Tregs was confirmed through the failure to suppress colitis in Rag2^-/-^ mice by the co-transfer of naïve T cells and Tregs. In lymphopenic or inflammatory conditions, TRAF6^-/-^ Tregs tended to lose Foxp3, and these cells were converted into Th2-like IL-4 producing cells. We propose that TRAF6 is essential for the maintenance of Tregs and suppression of IL-4 production from Tregs.

## Material and Methods

### Mice

Foxp3^Cre-YFP^ mice were kindly provided from Dr. Rudensky [18,19] and crossed with *TRAF6* ^flox/flox^ mice [16,20] to generate Treg specific *TRAF6* conditional knockout (cKO) mice. To fate map *Foxp3* in mice, *Foxp3* ^Cre-YFP^-*TRAF6* ^flox/flox^ (cKO) mice were crossed with R26tdRFP mice, which are knock-in mice carrying a tandem-dimer red fluorescent protein (tdRFP) within the Rosa26 locus [21]. Mice were kept in conventional conditions in Keio University (Tokyo, Japan). All experiments using these mice were approved by Institutional Animal Care and Use Committee (IACUC) (approved number 08004) of Keio University and performed according to the guidelines of IACUC. All experiments using these mice were approved by and performed according to the guidelines of the Animal Ethics Committee of Keio University.

### Histopathologic examination

Tissue samples were obtained from the proximal and distal colon, skin, liver, and kidney, and then fixed in 10% neutral buffered formalin, embedded in paraffin, then stained with hematoxylin and eosin (H&E) or Safranin-O as described previously [22,23].

### Treg suppression assay and transfer of Rag2^-/-^ mice

An *in vitro* suppression assay was conducted as described previously [13]. For the *in vivo* suppression assay, 4×10^5^ CD4^+^CD25^-^CD62L^+^CD44^-^ naïve T cells from WT mice purified with FACS were injected intravenously into Rag2^−/−^ mice in combination with 4 × 10^5^ CD3^+^CD4^+^CD25^+^Foxp3^Cre-YFP^ cells from WT (Foxp3^Cre-YFP^) mice or cKO mice as described [13]. Mice were observed and weighed daily. Four weeks after cell transfer, the mice were sacrificed, and sections of the colon were stained with H&E. Similarly, purified CD3^+^CD4^+^CD25^+^Foxp3^Cre-YFP^ cells from WT or cKO mice were injected intravenously into Rag2^−/−^ mice as described previously [13]. Mice were observed and weighed daily and they were sacrificed four weeks after cell transfer.

### Flow cytometry, cell sorting and cytokine secretion assays

Cell surface staining and flow cytometric analysis of CD3, CD4, CD25, CD62L and CD44 (all eBioscience) expression were performed as described previously [5]. For the isolation of Tregs, CD4^+^ T cells were positively selected using magnetic-activated cell sorting (Miltenyi), and CD3^+^CD4^+^CD25^+^Foxp3^Cre-YFP^ cells were further purified using a FACSAria cell sorter (Becton Dickinson). The purity of the sorted populations was invariably >99%. Intracellular staining of Foxp3 and IL-4 (all eBioscience) was performed following fixation and permeabilization according to manufacturer’s instructions [24]. To measure T cell cytokine production, cells were stimulated with PMA (50 ng/ml) and ionomycin (250 ng/ml) in the presence of Golgi Plug (BD Biosciences) for 4 hrs at 37°C before staining.

For STAT5 phosphorylation assay, Tregs were fixed with 4% paraformaldehyde for 10 min at 37°C, and then permeabilized by 90% MeOH (30 min on ice). Cells were then washed twice and stained with the Alexa Fluor®647-conjyugated anti-STAT5 antibody (BD Phosphoflow-STAT5pY694) and analyzed with a flow cytometer.

### Reverse transcription PCR analysis

Total RNA was prepared using a nucleospin RNA XS (MACHEREY-NAGEL). RNA was reverse-transcribed to cDNA with random primers (Applied Biosystems) and a high capacity cDNA reverse transcription kit in accordance with the manufacturer’s protocol (Applied Biosystems). To determine the cellular expression level of each gene, quantitative real-time PCR analysis was performed using a C1000 Thermal Cycler (BioRad). The PCR mixture consisted of 5 µl of KAPA SYBR FAST qPCR Kits (KAPABIOSYSTEMS), 15 pmol of forward and reverse primers, and the cDNA samples in a total volume of 10 µl. Relative RNA abundance was determined based on control *GAPDH* abundance. Primers were used as previously described [5,13].

### Enzyme-linked immunosorbent assay

ELISA assays for cytokines and immunoglobulins (Igs) were performed as per the manufacturer’s instructions (eBioscience). The optical density at 450 nm was determined using a Labsystems Multiscan MS (Analytical Instruments).

### Statistical analysis

All data were analyzed using a Student’s t test. The p-value of <0.05 was considered to be significant. All error bars shown represent standard deviations.

## Results

### Autoimmune phenotypes caused by Treg-specific TRAF6-deficient mice

Autoimmune phenotype of T cells specific TRAF6-cKO (CD4Cre-*TRAF6* ^fl/fl^) mice has been thought to be mostly caused by hyperactivation and Treg-resistance of effector T cells [16]. However, since Tregs from CD4Cre-*TRAF6* ^fl/fl^ mice showed normal suppression activity *in vitro*, functional alterations of TRAF6-deficient Tregs were not addressed further.

**Figure 1:**
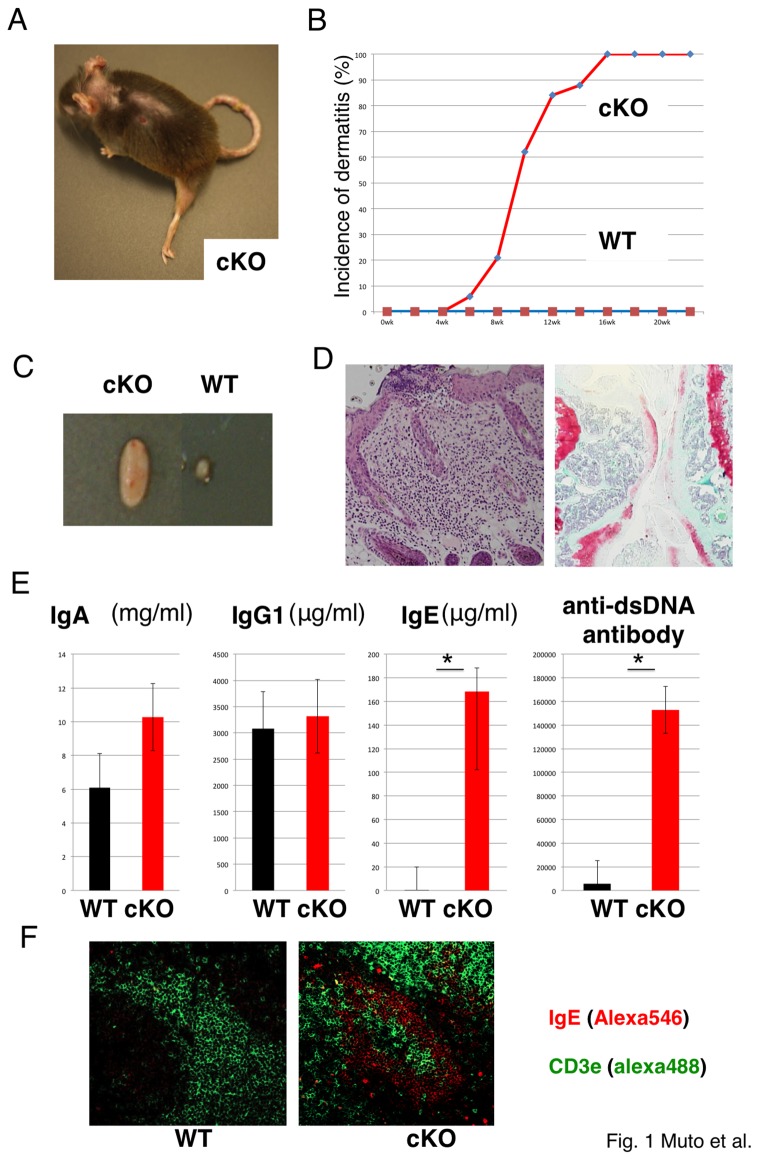
Characterization of *Foxp3* ^Cre-YFP^-*TRAF6* ^f/f^ (cKO) mice. (A) Representative appearance of a 22-week-old cKO mouse. (B) Incidence of dermatitis in cKO mice (*n* = 20). (C) Representative macroscopic observations of the LN from WT mice and age (about 20 weeks old)-matched TRAF6 cKO mice. (D) Representative histopathologies of the skin (H&E staining) and joint (Safranin-O staining) lesion of representative cKO mice. (E) Serum titers of immunoglobulin subclasses and anti-dsDNA antibodies from WT and cKO mice measured by ELISA. Each symbol indicates an individual host mouse (^*^ *p* < 0.05). (n=7) (F) Spontaneous splenic germinal center formation in cKO mice. Sections of spleen were stained with anti-IgE and anti-CD3 antibodies. Representative data form three independent mice are shown.

To examine the role of TRAF6 in Tregs, we generated Treg-specific TRAF6-cKO mice by using *Foxp3* ^Cre-YFP^ knock-in mice. This knock-in mouse harbors a cassette containing an internal ribosome entry site (IRES) followed by DNA sequence encoding a fusion protein (the yellow fluorescent protein (YFP)) with Cre recombinase [18]. Thus, Foxp3^+^ cells can be separated using YFP fluorescence. Surprisingly, similar to CD4Cre-TRAF6-cKO mice, dermatitis, splenomegaly, and lymph node (LN) swelling were observed in Treg-specific TRAF6 cKO mice, and these appeared in all the mice until they were 3 months of age (Figure **1A,B,C**). Arthritis and loss of cartilage tissues were observed in some mice (Figure **1D**). Although mononuclear cell infiltrates in the intestine, liver, lung and kidney were reported in CD4Cre-TRAF6^fl/fl^ mice, we did not observe strong inflammation in these organs. We observed a high increase of IgE as well as anti-dsDNA antibody, but not total IgG1and IgA in the serum, suggesting Th2-type autoimmunity (Figure **1E**). We observed spontaneous germinal center formation in the spleen of Foxp3^Cre-YFP^-TRAF6^fl/fl^ mice (Figure **1F**). These diseases were more severe in female mice than in male mice. Thus, Treg specific TRAF6 deletion resulted in allergy and SLE-like autoimmunity, although the diseases were slightly milder than in whole T cell-specific TRAF6 deletion.

### Th2-type hyperactivation of CD4^+^T cells in TRAF6-cKO mice

**Figure 2:**
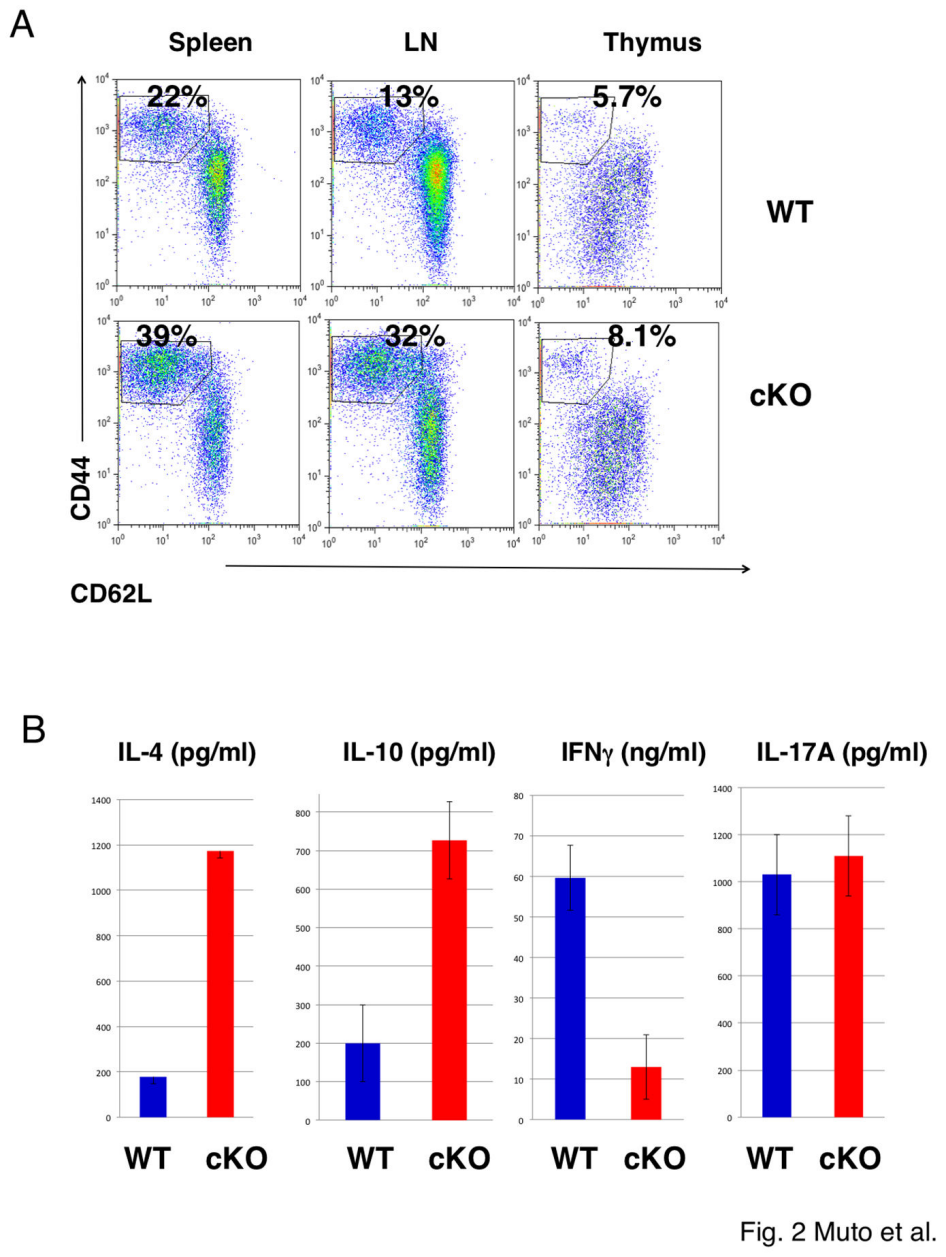
Activation of T cells in Treg-specific TRAF6-cKO mice. (A) Flow cytometry for activated/memory phenotypes of freshly isolated CD4^+^ T cells from thymus, spleen and mesenteric lymph nodes (MLNs) in the indicated mice at 22 weeks of age. Representative data from three independent mice are shown. (B) Cytokine production from splenic T cells. Freshly isolated splencytes (1x10^6^) from WT and cKO mice were stimulated with anti-CD3 antibody for 2 days. Cytokine levels in the culture supernatant was measured using ELISA (n=3).

We also examined T cell activation. As shown in Figure **2A**, a substantial fraction of effector-memory (CD44^high^, CD62L^low^) CD4^+^ T cells were increased in the spleen and LN, but not so highly in the thymus, of cKO mice. In addition, we examined cytokine production from the splenic T cells. As shown in Figure **2B**, splenic T cells from cKO mice produced higher levels of IL-4 and IL-10, and lower levels of IFN-γ in response to TCR stimulation compared to T cells from WT mice. IL-17 levels were not altered between WT and cKO mice. These data suggested that effector T cells were skewed into Th2-type differentiation and hyperactivated at the periphery.

### Functional defects of TRAF6-deficient Tregs

**Figure 3:**
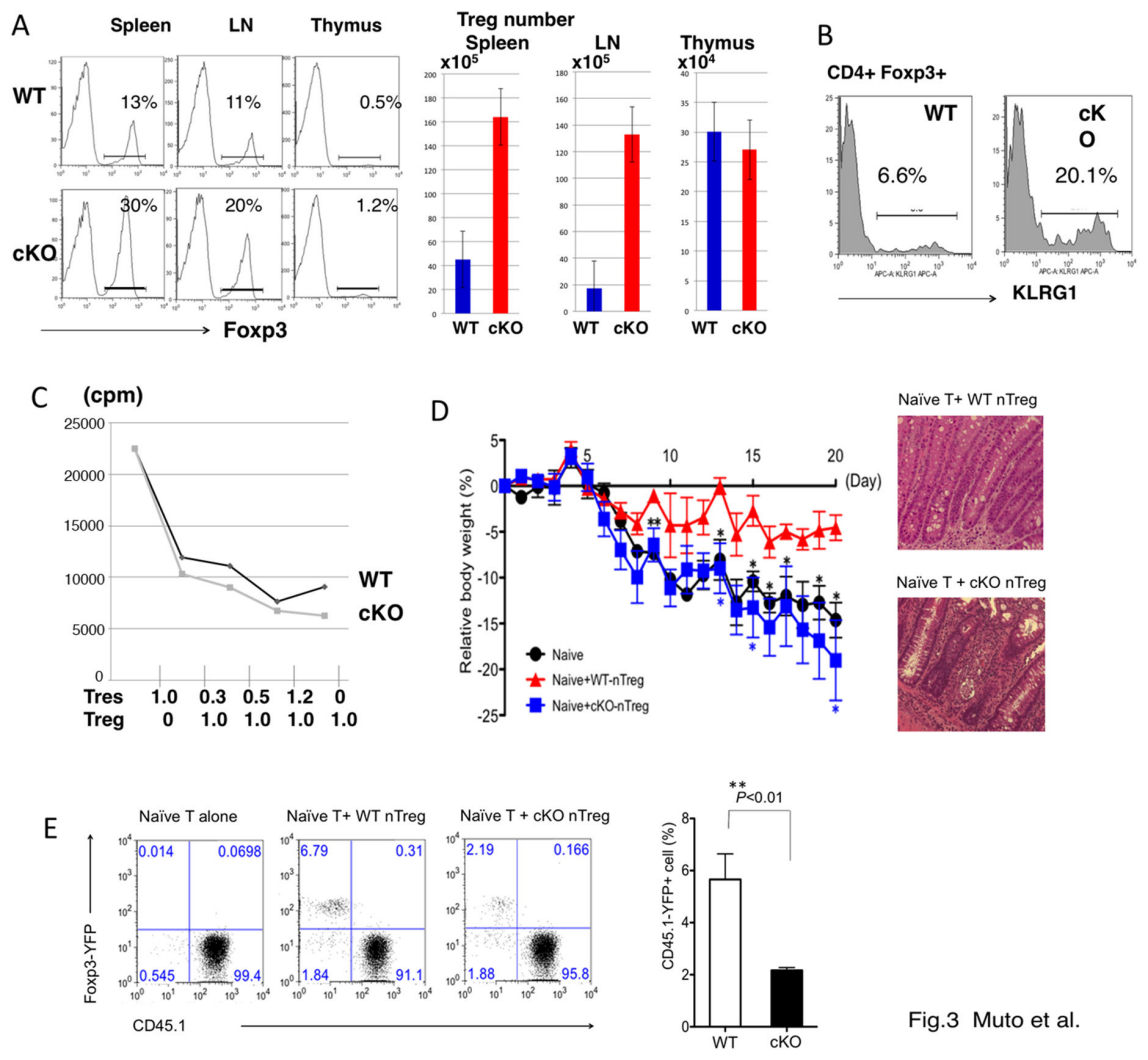
Reduced stability of Foxp3 in *TRAF6*-deficient Treg cells (A) Foxp3 expression in freshly isolated CD4^+^ T cells from the spleen, mesenteric lymph node (LN) and thymus of the indicated mice. Foxp3 levels were measured using YFP-fluorescence. Data are representative of five independent experiments with similar results. The numbers of YFP^+^ Tregs in each organ are shown in right graphs (n=5). (B) Expression of KLRG1 was measured in CD4^+^Foxp3(YFP)^+^ gated cells. (C) *In* *vitro* suppression assay. FACS purified 5×10^4^ CD4^+^CD25^-^CD62L^+^CD44^-^ naïve T cells from WT mice were stimulated for 72 hours with 10^5^ T-cell depleted spleen cells and anti-CD3 (2C11) (1µg/ml), in the absence or presence of CD3^+^CD4^+^Foxp3(YFP) + Treg cells from WT (diamond) or cKO (square) mice as indicated concentrations. Cells were pulsed with 0.5 µCi of ^3^H-thymidine for the final 16 hours before being harvested. Error bars indicate SD; n=3. (D) *In* *vivo* suppression assay. 3 x 10^5^ naïve T cells from the LN of CD45.1^+^ WT mice together with or without 1.5 x 10^5^ Tregs (CD4^+^YFP ^+^ RFP^+^) from the LN of CD45.2 Foxp3 ^YFP-Cre^ROSA26^RFP^ TRAF6^+/+^ (WT-Tregs) or CD45.2 Foxp3 ^YFP-Cre^ROSA26^RFP^ TRAF6^fl/fl^ (cKO-Tregs) were transferred into Rag2^-/-^ mice. Body weight changes were measured daily. Data are representative from four independent experiments. *p<0.05 **p<0.01; Representative histology of the colon is shown in right panels. Magnification x200 (E) Flow cytometric analysis of Foxp3-YFP and CD45.1 expression on CD3^+^CD4^+^ T cells from the LN in Rag2^-/-^ mice. Data are representative from four independent experiments. Quantification of the data of the fraction of YFP ^+^ CD45.1^-^ cells (remaining Foxp3 ^+^ Tregs) is shown in the right panel. **p< 0.01.

We also examined the phenotypes of Tregs in cKO mice. The number of Foxp3 ^+^ Tregs was 2 to 3 times higher in the spleen and LN, but not in the thymus, of cKO mice than in WT mice that were 8 to 20 weeks old (Figure **3A**). KLRG1 has been shown to be a marker of activated Tregs [25]. As shown in Figure 3A, KLRG1^+^ Tregs were increased in the CD4^+^Foxp3(YFP)^+^ fraction in cKO mice compared to WT mice (Figure **3B**). These data suggested that Foxp3^+^ Tregs in cKO mice likely expanded and activated in the periphery under inflammatory conditions.

Then, we examined the suppression function of TRAF6-deficient Tregs. TRAF6-deficiency did not affect *in vitro* suppression activity (Figure **3C**). For *in vivo* suppression assay, naïve T cells (CD45.1^+^) were co-transferred with Tregs (CD45.2^+^) into Rag2^-/-^ mice (Figure **3D**). To obtain highly purified Tregs, we crossed Foxp3^Cre-YFP^-TRAF6^fl/fl^ mice with reporter mice that express red fluorescent protein (RFP) from the Rosa26 promoter only after excision of a loxP-flanked stop cassette (Rosa26-loxP-StoploxP-RFP (R26-RFP) mice) [12]. Resulting mice were so-called fate mapping mice. Tregs were purified using FACS sorting as CD3^+^CD4^+^ YFP ^+^ RFP^+^ (>99% Foxp3-positive) from the LN of CD45.2^+^ Foxp3 ^YFP-Cre^ROSA26^RFP^ TRAF6^+/+^ (WT-Tregs) or CD45.2^+^ Foxp3 ^YFP-Cre^ROSA26^RFP^ TRAF6^fl/fl^ (cKO-Tregs). Body weight changes were measured daily. Body weight loss resulting from colitis was observed in Rag2^-/-^ mice that received a transfer of naïve CD4^+^T cells. Co-transfer of naïve T cells together with WT Tregs resulted in the suppression of colitis and body weight gain (Figure **3D** **, naïve T+WT Treg**). In contrast, when naïve T cells were co-transferred with TRAF6^-/-^Tregs, Rag2-deficient mice showed colitis (Figure **3D**, naïve T+cKO Treg) like without Tregs (naïve T cell alone). Histological examination also confirmed infiltration of mononuclear cells in TRAF6-deficeint Treg-transferred mice but not in WT Treg-transferred mice (Figure **3D**, right panels). These data indicate that suppression activity of TRAF6^-/-^ Tregs was impaired *in vivo*.

To determine the fate of transferred Tregs, which were CD45.2-positive, we examined Foxp3-positivity using CD45.1 and CD45.2 markers. In this co-transfer condition, about 20-30% of WT Foxp3 ^+^ CD45.1^-^ Tregs become Foxp3-negative 4 weeks after transfer, and Foxp3 ^+^ Tregs remained at a level of approximately 6% of the total CD4^+^ T cells (Figure **3E**). However, TRAF6^-/-^Tregs did not expand well and the fraction of Foxp3 ^+^ Tregs in whole CD4^+^ T cells was less than 2%. The ratio of Foxp3^-^ cells (exFoxp3 cells) among CD45.2^+^ cells was approximately 40-50%. These data suggest that TRAF6 in Tregs plays an important role in the expansion and/or maintenance of Tregs under lymphopenic conditions in the presence of effector T cells.

### Defects in the expansion and maintenance of Tregs by TRAF6-deficiency

**Figure 4:**
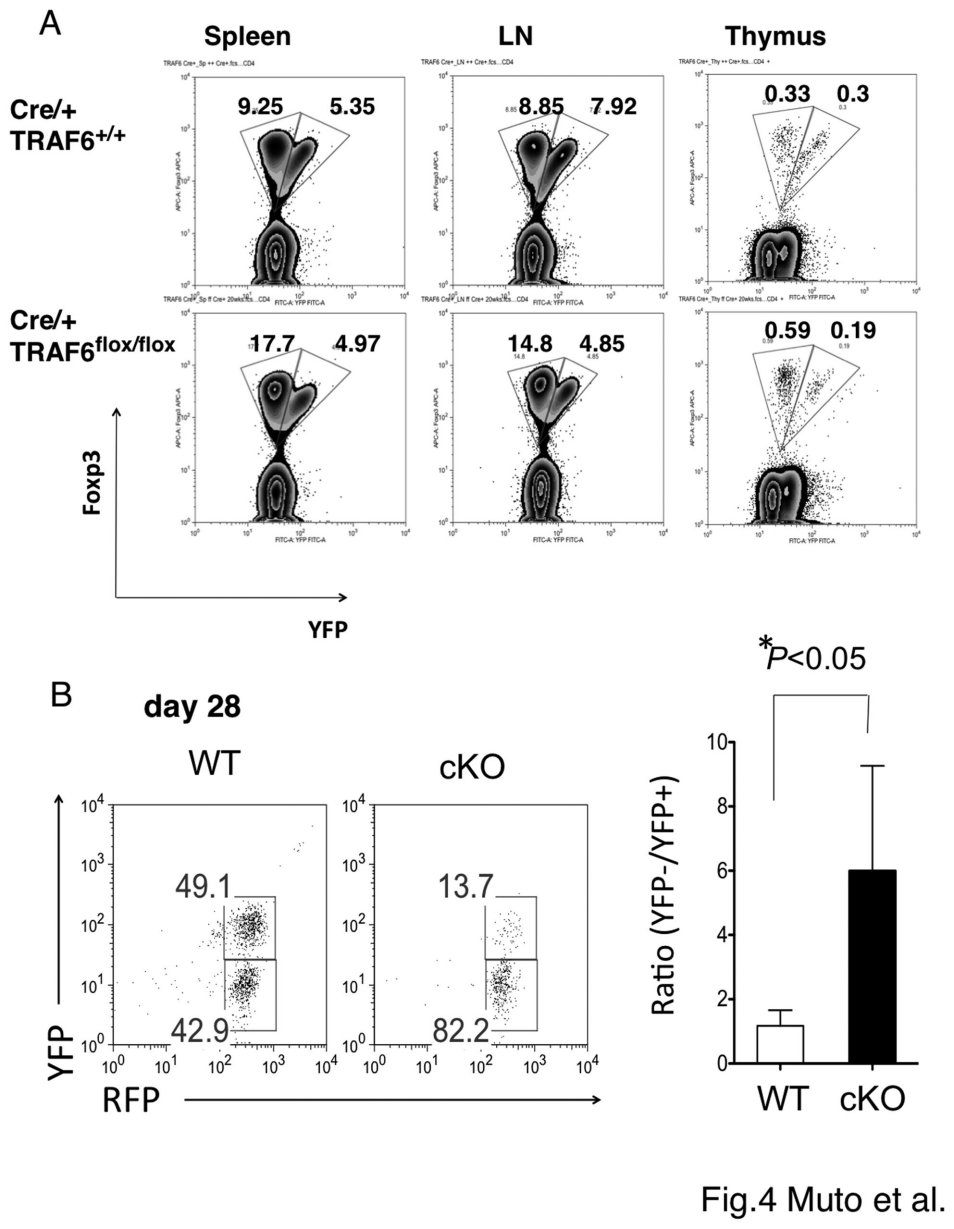
Reduced expression of Foxp3 in TRAF6-deficeit Tregs. (A) Foxp3 protein and YFP expression in Treg cells among gated CD4^+^T cells from the spleen, LN and thymus of female Foxp3 ^YFP-Cre/+^ TRAF6^f/f^ mice and Foxp3 ^YFP-Cre/+^ TRAF6^+/+^ littermates. Numbers adjacent to the outlined areas indicate percent Foxp3 ^+^ YFP^-^ cells (top left) or Foxp3+YFP+ cells (top right). These results are representative of three similar experiments. (B) Loss of Foxp3-positivity in lymphopenic conditions. 2 x 10^5^ CD4^+^Foxp3(YFP ^+^ RFP^+^) cells from WT (Foxp3 ^YFP-Cre^ROSA26^RFP^ TRAF6^+/+^) or cKO (Foxp3 ^YFP-Cre^ROSA26^RFP^ TRAF6^fl/fl^) mice were transferred Tregs into Rag2^-/-^ mice. Four weeks after transfer, Foxp3-positivity in CD4^+^RFP^+^ cells was measured. Raito of YFP-/YFP+ is shown in the right panel (N=3). *p<0.05.

To compare the number of TRAF6^+/+^ and TRAF6^-/-^ Tregs in the same mice under non-inflammatory conditions, we examined the fraction of both Treg genotypes in a single female Foxp3 ^Cre/+^ TRAF6^fl/fl^ mouse. Since the Foxp3 gene resides in the X-chromosome, Foxp3 ^Cre/+^ TRAF6^fl/fl^ female mice possessed both TRAF6-positive and negative Tregs due to random X-chromosome inactivation. Thus, the YFP ^+^ Foxp3^+^ : YFP^-^Foxp3^+^ ratio must be 1:1 in wild type female Foxp3 ^Cre/+^ TRAF6^+/+^ mice. indeed, the YFP ^+^ /YFP^-^ ratio in TRAF6^+/+^ (WT) females was near 1:1 in both the thymus and the periphery (Figure **4A**). However, the YFP ^+^ /YFP^-^ ratio in Foxp3^Cre/+^ TRAF6^fl/fl^ females was 1:3 in the thymus and 1:4 in the periphery, indicating that intrinsic TRAF6 in Tregs are necessary for the proper proliferation or expansion of Tregs.

Next, to examine the stability of Tregs under lymphopenic conditions, we transferred Foxp3^+^ (YFP ^+^ RFP^+^ cells from fate mapping mice) cells into *Rag2*-deficient mice, and compared the homeostatic expansion and Foxp3 expression in Tregs. As shown in Figure **4B**, the fraction of Foxp3^-^ cells (exFoxp3 cells), which lost Foxp3 expression from Tregs was much higher in Rag2^-/-^ mice with TRAF6-deficient Tregs transfer than in Rag2^-/-^ mice with WT Tegs transfer. These data together with Figure **3E** data suggest that TRAF6 plays an important role not only in the expansion but also in stability of Tregs. In the absence of TRAF6, loss of Foxp3 expression in Tregs, or conversion to exFoxp3 cells was apparently accelerated.

### Loss of Foxp3 expression and conversion to Th2-like cells for TRAF6-deficient Tregs in vivo

**Figure 5:**
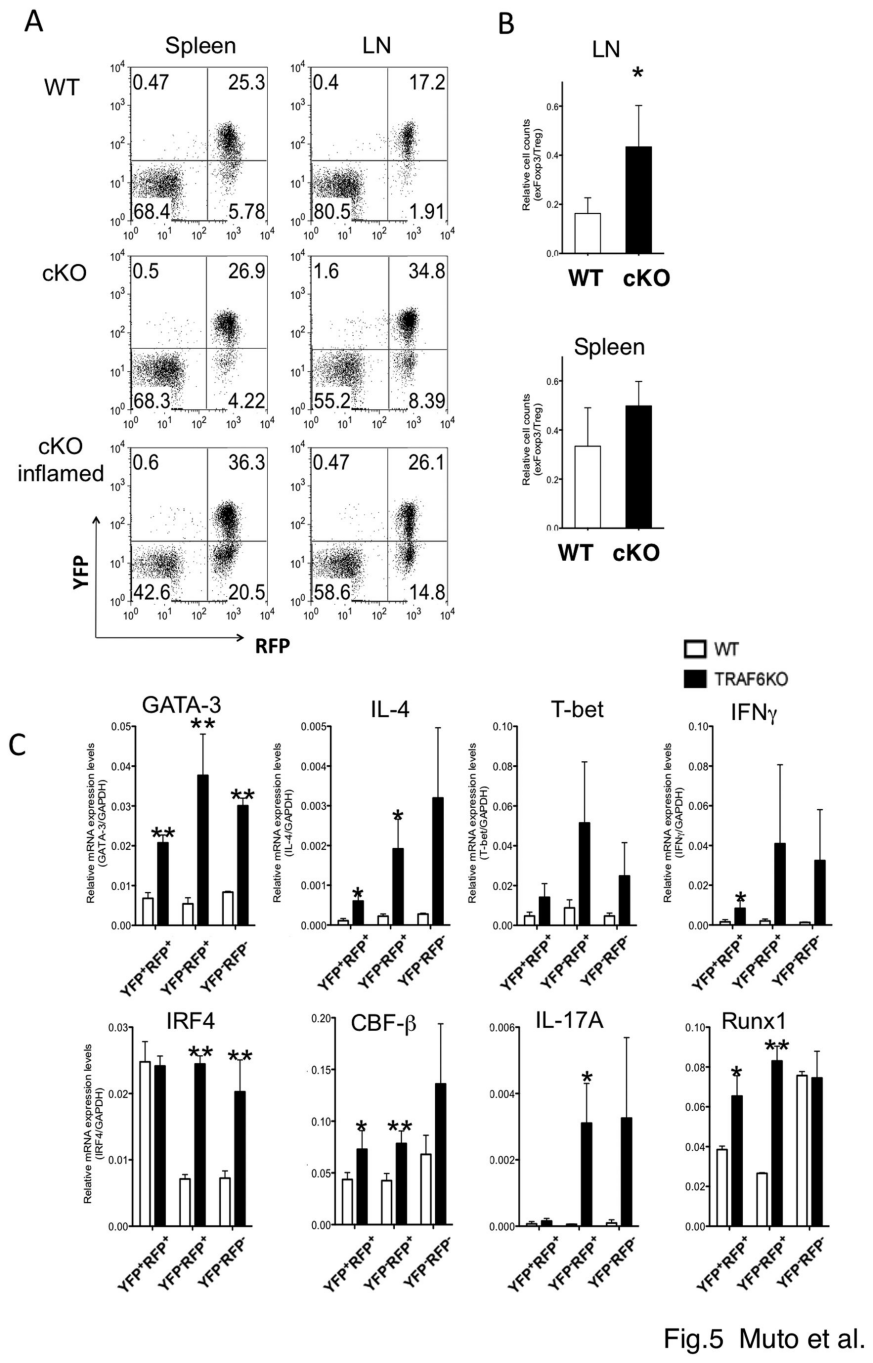
Fate mapping study of *TRAF6*-deficeint Tregs. (A) FACS profiles of CD4+ T cells in the spleen of WT (Foxp3 ^YFP-Cre^ROSA26^RFP^ TRAF6^+/+^) or cKO (Foxp3 ^YFP-Cre^ROSA26^RFP^ TRAF6^fl/fl^) mice. A representative profile of WT and cKO mice (16-20 weeks old) with or without severe inflammation. (B) RFP ^+^ YFP^-^/RFP ^+^ YFP^+^ ratio in the spleen of WT and cKO mice at 12 to 22 weeks of age (n=5). (C) mRNA levels in each CD4^+^T cell fraction. After sorting using FACS with YFP and RFP, mRNA was isolated from each fraction, and the levels of indicated genes were measured using quantitative real time RT-PCR. The mean ± SD of three independent experiments are shown. All data were analyzed using a Student’s t test. *p<0.05, **p<0.01vs. WT.

We then investigate the fate of Tregs *in vivo* by using fate mapping mice. As shown in Figure **5A**, there is an exFoxp3 (YFP^-^RFP^+^) cell fraction in WT CD4^+^ T cells, as reported previously [10]. This fraction is increased in TRAF6^-/-^ mice, which is accompanied by an increase in Tregs (YFP ^+^ RFP^+^). Furthermore, the ratio of exFoxp3:Treg was slightly higher in TRAF6 cKO mice than in WT mice, especially in the LN, (Figure **5B**), supporting that loss of Foxp3 was accelerated by TRAF6-deficiency. We observed a high increase in the exFoxp3:Treg ratio in cKO mice with severe inflammation (Figure **5A**), and thus conversion from Tregs to exFoxp3 cells seemed to be accelerated by TRAF6-deficiency under inflammatory conditions.

To determine whether TRAF6-deficient exFoxp3 is pathogenic, we examined the expression of cytokines in this fraction. As shown in Figure **5C**, exFoxp3 cells from TRAF6^-/-^ mice (YFP^-^RFP ^+^ CD4^+^ T cells) as well as conventional T cells (YFP^-^RFP^-^) expressed higher levels of GATA3 and IL-4 than those from WT mice. We observed slight increase of GATA3 and T-bet even in TRAF6-deficeint YFP ^+^ RFP^+^ Tregs than in WT YFP ^+^ RFP ^+^ Treg. *IFN-γ* and *T-bet* levels were also increased in TRAF6-deficient T cells, however, differences were not statically significant (Figure **5C**). IL-17 was also detected in TRAF6-deficeint exFoxp3 fraction. These data also suggest that TRAF6-deficient Tregs were ready to express cytokines and to become effector cells after loss of Foxp3. These higher levels of IL-4 from TRAF6-deficient exFoxp3 cells may contribute to the higher expression of IL-4 from effector T cells (YFP^-^RFP^-^ in Figure **5C**) of TRAF6-cKO mice.

### Similar proliferation and STAT5 activation between WT and TRAF6-deficient Tregs in vitro

**Figure 6:**
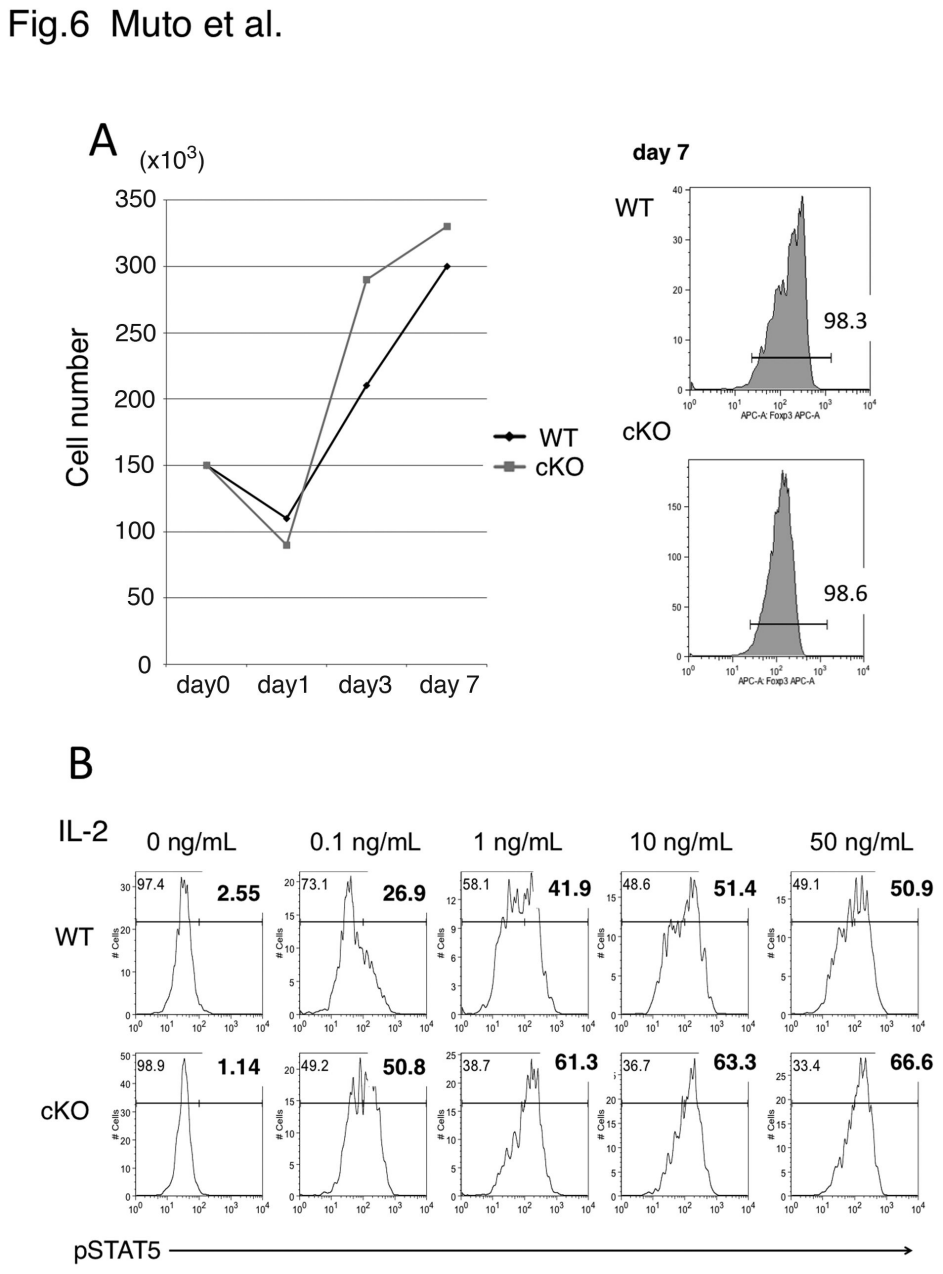
IL-2 responses of WT and TRAF6-deficeint Tregs. (A) CD4^+^CD25^+^YFP^+^ cells were isolated by FACS from the spleen and LN of male Foxp3 ^YFP-Cre^TRAF6^f/f^ mice and Foxp3 ^YFP-Cre^TRAF6^+/+^ mice, and 1.5x10^5^ cells were cultured with plate-bound anti-CD3 antibody (1 μg/plate) and CD28 (1 µg/ml) in the presence of 25 ng/ml IL-2. The number of viable cells was counted after culture of indicated days. YFP-positivity was measured on day 7 (right panels). Representative data of three independent experiments. (B) STAT5 phosphorylation. Freshly isolated CD4^+^YFP^+^ cells were stimulated with indicated concentrations of IL-2 for 40 min. Then cells were stained with an anti-STAT5 (pY694) antibody and analyzed with a flow cytometer. Representative data from three independent experiments is presented.

To determine whether reduced Foxp3 ^+^ T cells by TRAF6 deficiency in female cKO mice and in lymphopenic conditions, we examined the proliferation capacity of Tregs *in vitro*. FACS-purified Tregs were stimulated with anti-CD3/anti-CD28 antibodies in the presence of IL-2. As shown in Figure **6A**, Treg proliferation was similar between WT and TRAF6-deficeint Tregs. We also could not find any difference in Foxp3 levels between WT and TRAF6-deficient Tregs in these conditions (Figure **6A**, right panels). Cell death was not significantly different between WT and TRAF6-deficeint Tregs (data not shown). Since STAT5 activation has been shown to be critical for Treg expansion and survival, we then examined STAT5 activation in response to various concentrations of IL-2 (Figure **6B**). STAT5 phosphorylation was rather stronger in TRAF6-deficeint Tregs than in WT Tregs. Thus, these data suggest that TCR and IL-2 signals in Tregs were not altered by TRAF6-deficiency at least *in vitro*.

## Discussion

Our present study demonstrates that TRAF6 plays an important role in Treg expansion and/or Treg stability. TRAF6-deficiency lead a rapid conversion of Foxp3 ^+^ Tregs to Foxp3^-^ exFoxp3 cells in lymphopenic conditions (Figure **4B**). TRAF6-deficient Foxp3+Treg cells tended to express higher GATA3 and IL-4 before losing Foxp3, and the levels of these factors further increased in exFoxp3 cells (Figure **5C**). We also observed an increase of T-bet, IRF4, IFN-γ and IL-17 in TRAF6-deficeint exFoxp3 cells. These data seems to be contradictory to Th2-prone phenotypes of TRAF6-cKO mice as shown in Figure **2**. We could not detect IFN-γ and IL-17 at high levels in the spleen of cKO mice probably because we used mice with established disease in Figure **2**. Although the precise mechanism is not clear at present, TRAF6-deficeint exFoxp3 cells seem to possess highly activated phenotypes, and Th2 character may become predominant *in vivo* as the disease progresses.

Although the importance of TRAF6 signaling for T cell proliferation and survival has been demonstrated, the exact mechanism of how TRAF6 signaling supports Treg proliferation *in vivo* remains to be clarified. TRAF6-deficient T cells were hypersensitive to IL-2 and CD28 signaling [16,17], and thus, such reduced proliferation of TRAF6-deficient Tregs *in vivo* could occur for other reasons. Recently, Cejas et al reported that TRAF6 deficiency renders effector T cells more sensitive to TGF-β-induced Smad2/3 activation and proliferation arrest [26]. Consistent with this, in TRAF6-deficient T cells, TGF-β more effectively down-regulates IL-2, which is an important factor for Treg expansion. Therefore, a possible mechanism for the low expansion of TRAF6-deficient T cells is reduced IL-2 sensitivity of the TRAF6-deficient Tregs. However, in *vitro*, we could not find any defects in proliferation and IL-2 signaling in TRAF6-deficient Tregs (Figure 6). Thus, reduced sensitivity to IL-2 may not the case in TRAF6-deficeint Tregs, although we could not rule out a possibility of the effect of TGF-β *in vivo* situation. We could not provide clear molecular mechanism at present. Further study is necessary to clarify how TRAF6 functions in Treg stability and expansion *in vivo*.

TRAF6 has been shown to play an essential role in activating NF-kB in T cells, and recent studies indicate that it also plays a critical role in the development of Treg cells [27-29]. Two NF-kB proteins, c-Rel and p65, drive the development of Treg cells by promoting the formation of a Foxp3-specific enhanceosome. c-Rel has been shown to bind to the Foxp3 enhancer region, which is specifically demethylated in nTreg cells. Consequently, c-Rel-deficient mice have marked reductions in Treg cells, and c-Rel-deficient T cells are compromised in Treg cell differentiation [30]. Thus, one possibility is that the reduced NF-kB activity in TRAF6^-/-^Tregs affected induction of Foxp3 during Treg development. TRAF6 is also a downstream of IL-1 and TLR family receptors. It has been shown that several TLRs are expressed in Tregs and functions *in vivo* [31]. Thus, further study for IL-1/TLR signals in Tregs may improve the understanding of the role of TRAF6 in Tregs.

It has been shown that the fraction of exFoxp3 increases under inflammatory conditions [13]. In lymphopenic conditions, about half of the transferred Tregs converted to Foxp3-negative cells that express IL-17 or IFN-γ [13] or differentiated into Tfh cells in Peyer’s patches [9]. Treg-specific deletions of *IRF4* [32] and *Cbf*β [33] resulted in Th2-type inflammatory diseases such as lymphoproliferation, autoimmune disease in the lung and skin, and hyperproduction of IgE. Treg-specific TRAF6 cKO mice developed similar Th2-type diseases and a similar increase in IL-4 expression in Tregs. IRF4 and/or Runx-Cbfβ could be downstream of TRAF6 signaling. However, we did not find any decrease of IRF-4, Runx1 and Cbfβ in TRAF6-deficeint Tregs (Figure **5C**). These factors were rather higher in TRAF6-deficeit exFoxp3 cells. Thus these may rather be involved in higher production of cytokines from exFoxp3 cells.

Impaired Treg function has been shown to be associated with human autoimmune diseases including rheumatoid arthritis (RA) and multiple sclerosis (MS). Recently, Nie et al showed that Tregs from RA patients possessed reduced suppression activity due dephosphorylation of Foxp3 by TNF-α, which is high in human RA patients [34]. TNF-α-induced Treg cell dysfunction correlated with increased numbers of Th1 and Th17 cells within the inflamed synovium in rheumatoid arthritis. Another study showed the presence of IFN-γ ^+^ Foxp3^+^ T cells in MS patients and these double positive cells acquired a Th1-like phenotype and reduced suppression activity when cultured in the presence of interleukin-12 [35]. Since recent report revealed that TRAF6 is associated with RA and SLE patients [36,37], TRAF6 and its signaling molecules could be responsible for Treg dysfunction in human immune disorders.
